# Tagged MEG measures binocular rivalry in a cortical network that predicts alternation rate

**DOI:** 10.1371/journal.pone.0218529

**Published:** 2019-07-11

**Authors:** Elizabeth A. Bock, Jeremy D. Fesi, Sylvain Baillet, Janine D. Mendola

**Affiliations:** 1 Neurology and Neurosurgery, McGill University, Montreal, Quebec, Canada; 2 McConnell Brain Imaging Centre, Montreal Neurological Institute, Montreal, Quebec, Canada; 3 Department of Ophthalmology, McGill University, Montreal, Quebec, Canada; Monash University, AUSTRALIA

## Abstract

Binocular rivalry (BR) is a dynamic visual illusion that provides insight into the cortical mechanisms of visual awareness, stimulus selection, and object identification. When dissimilar binocular images cannot be fused, perception switches every few seconds between the left and right eye images. The speed at which individuals switch between alternatives is a stable, partially heritable trait. In order to isolate the monocular and binocular processes that determine the speed of rivalry, we presented stimuli tagged with a different flicker frequency in each eye and applied stimulus-phase locked MEG source imaging. We hypothesized that the strength of the evoked fundamental or intermodulation frequencies would vary when comparing Fast and Slow Switchers. Ten subjects reported perceptual alternations, with mean dominance durations between 1.2–4.0 sec. During BR, event-related monocular input in V1, and broadly in higher-tier ventral temporal cortex, waxed and waned with the periods of left or right eye dominance/suppression. In addition, we show that Slow Switchers produce greater evoked intermodulation frequency responses in a cortical network composed of V1, lateral occipital, posterior STS, retrosplenial & superior parietal cortices. Importantly, these dominance durations were not predictable from the brain responses to either of the fundamental tagging frequencies in isolation, nor from any responses to a pattern rivalry control condition, or a non-rivalrous control. The novel cortical network isolated, which overlaps with the default-mode network, may contain neurons that compute the level of endogenous monocular difference, and monitor accumulation of this conflict over extended periods of time. These findings are the first to relate the speed of rivalry across observers to the ‘efficient coding’ theory of computing binocular differences that may apply to binocular vision generally.

## Introduction

Binocular rivalry (BR) is a well-known example of bistable visual perception that has been studied intensely from both behavioral and neuroimaging perspectives [1–4]. The interest in this phenomenon stems from multiple factors. Like most visual illusions, BR provides a dissociation between the retinal input (i.e., constant incompatible images shown to each eye), and dynamic conscious perception that switches spontaneously between the two alternatives. Equally important to vision science is the opportunity to observe and manipulate reciprocal inhibitory influences between the two eyes. In fact, the precise rate at which perception switches between the alternatives appears to be a sensitive measure of interocular competition, with greater inhibition generally leading to slower rivalry, e.g., [4,5]. This rate of alternation (and by extension strength of competition) is modulated systematically *within* subjects as the stimuli change, e.g., the contrast of grating stimuli, or the between-eye difference in orientation, e.g., [6–8]. In addition, BR alternation rate also varies *between* subjects who view the same stimulus [9–12,5]. Previous twin-studies suggest that such variation may be partially heritable [13]. There is also some indication that this trait might be altered in autism or bipolar disorder, as well as with aging [14–17]. Nevertheless, many questions remain about the relevant mechanisms.

Brain imaging methods can provide neural correlates of such individual differences, and powerful opportunities to better understand the functional mechanisms involved [18,19]. A couple of suggestive studies have found neurophysiological indices that relate to individual strength of interocular inhibition using fMRI or magneto-encephalography (MEG) [20,21]. In addition, there is some evidence that the evoked gamma-band (40–80 Hz) MEG response to simple (nonrivalrous) stimuli provides a proxy measure of local resting tonic inhibition in primary visual cortex, measured via inhibitory neurotransmitter GABA levels obtained with MR spectroscopy [22–24]. Interestingly, this potential MEG proxy measure of GABA concentrations in V1 also correlates with individual binocular rivalry rates [25,26].

Thus, the literature as a whole indicates that subtle variations in the balance of excitation and inhibition in visual cortex might help to explain the variation in rivalry alternation rate between subjects. However, gaps in knowledge remain. None of the previous studies provided full brain coverage while localizing effects to specific visual areas. Equally important is the need to separate the role of monocular and binocular neurons in producing these effects. This level of detail is critical to distinguish between different computational models of binocular rivalry, e.g., [27–30].

We wished to fill these gaps using tonic presentations of frequency-tagged visual stimuli, inducing brain steady-state visual-evoked responses (SSVERs) captured with MEG source imaging. The rationale for tagging is that each monocular input to visual cortex can be separated by presenting stimuli in the left and right eye with a different flicker frequency. Multiple studies have shown that the SSVER to the monocular tagging frequency of the left and right eye varies systematically according to the dominance and suppression phases reported by individual subjects via button press [31–35]. However, the strength and location of this competition throughout the brain is controversial and unclear. Moreover, the specific role of binocular neurons is unknown. One way to isolate binocular neurons is to study time-resolved cortical activity at *intermodulation* frequencies of the tagged visual monocular stimuli, which are hallmarks of nonlinear binocular combination [36,37,31]. Such intermodulation frequencies could result from either summation or differencing between the fundamentals or their harmonics. Interestingly, recent studies have linked such intermodulation frequencies to perceptual conditions of high interocular conflict [38,39]. A good candidate mechanism to explain these effects involves *ocular opponent neurons*, for which firing rate increases with the difference (i.e., conflict) between the two eyes’ stimuli [30], but physiological evidence is limited.

**Fig 1:**
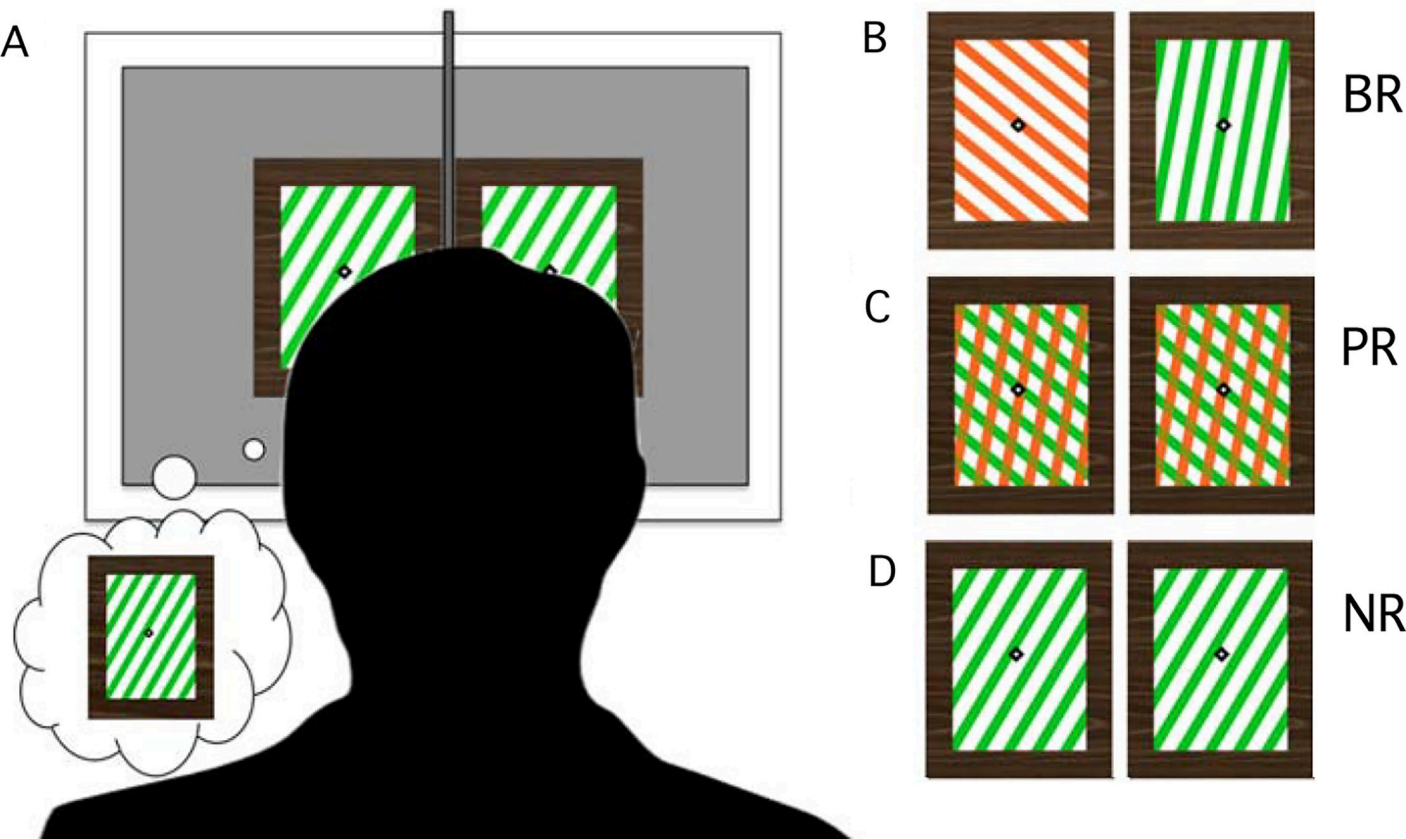
Dichoptic viewing setup and three task conditions. A. Subjects wore prism glasses that allowed fusion of separate stimuli viewed by the left or right eye. An opaque divider was placed between the subject’s face and the screen to block any additional images and scattered light. Three tasks with similar but different stimuli were used. In all cases, the stimulus shown to the left eye flickered at 5 Hz, and to the right eye at 7.5 Hz. B. Binocular rivalry (BR) used incompatible images in each eye that different in color and orientation. Subjects indicated their perceptual alternations with button press. C. Pattern rivalry control task (PR) used identical stimuli in each eye. However, the two grating components (with different color and orientation) alternated in visibility/salience, and subjects reported their alternations with button press. Note here that eyes not grating components were tagged. D. No rivalry (NR) was a type of replay control task with identical stimuli in each eye that changed physically every 1–3 seconds to mimic natural rivalry.

In sum, the previous body of work suggests that individual differences in the speed of rivalry correlate with resting levels of inhibition, and that this might further be related to levels of cortical interocular competition. In the present study, we hypothesized more specifically that, during BR, magnitude measures of oscillatory brain activity at the fundamental and intermodulation frequencies of tagged binocular stimuli, as signal markers of eye-specific interactions, and binocular integration respectively, might also reveal individual differences. We also build on our previous results with fMRI [40] by including a pattern rivalry (PR) control condition. PR differs from BR in that the left and right eye images are identical, and yet a bistable percept ensues because visual attention shifts between different stimulus features (**Fig 1**). Nevertheless, in PR the suppression is weaker (alternations of stimulus clarity not visibility) and competition likely occurs only between binocular neurons. Therefore, we hypothesized little or no monocular competition would be evident for fundamental frequencies tagging the left and right eye, despite the ongoing rivalry alternations. Similarly, no such competition was expected for a matched *non-rivalrous* control condition, despite tagging the left and right eye. We also expected that effects might vary systematically between slow and fast switchers, even though our sample size of 10 is modest for assessing individual differences.

## Materials and methods

Ten subjects, 5 females, with mean age 25 were recruited via a participant list at McGill University’s McConnell Brain Imaging Centre and consented to participate in the study. They were all tested for normal or corrected-to-normal vision using a Snellen Optotype Acuity test and a stereoacuity test. All participants were also tested for compatibility with the MEG system and were provided time for practice runs to ensure they could properly perform the task. The experiments were approved by the Research Ethics Board of the Montreal Neurological Institute (Protocol NEU-12-049), and all participants provided written informed consent.

### Stimuli and task

The stimuli were presented using MATLAB (2012b) and Psychophysics Toolbox (version 3.0.10) [41–43], with a Sanyo-PLC projector with a 60-Hz refresh rate [25]. The stimuli consisted of two images, one presented to the right eye, and one to the left. For all conditions, the stimulus shown to the left eye flickered at 5 Hz, and to the right eye at 7.5 Hz. All stimuli were gratings with spatial frequency approximately 3 cpd and were presented for fusion via prism lenses. Once fused, the grating subtended 9.5 x 13 deg of visual angle from the center of the screen. The use of colored stimuli was necessary in order to create robust rivalry for PR with alternation rates similar to that of BR [44]. For the sake of compatibility with the PR task, we used chromatic gratings for all tasks. As displayed by the facility projector, the green gratings (with CIE 1931 xy coordinates of: 0.33875, 0.60425) had a contrast of 42% against the white components of the stimuli (luminance: 657.55 c/m2), and the red gratings (CIEx,y: 0.5337, 0.42175) had a similar contrast of 47%. The gratings were presented in pairs that differed 60 deg in orientation: 50/350deg, and 30/330deg with the color of each orientation and left-versus right eye position counterbalanced and changing every trial, to prevent color and classical adaptation.

The subjects participated in three tasks: binocular rivalry (BR), pattern rivalry (PR) and a "replay" task with similar sensory input and motor response, but lacking visual competition, referred to as *no rivalry* (NR) (Fig 1). Stimuli for the BR task were gratings that differed in orientation between eyes by 60 deg (left oblique versus right oblique). The PR stimuli consisted of similar overlaid gratings that formed a plaid, consisting of 50/350deg, or 30/330deg orientation pairs. The gratings were red versus green against a white background. The color of the grating intersections was defined as the product of the red and green color values. The intersections were consistent with a percept of transparency, for which the subject would perceive one color or the other. The NR stimulus was the same in each eye but varied between a red left- or right-oblique and a green left- or right-oblique. The NR switch was presented with randomly jittered inter-stimulus interval (ISI) between 1.5 and 3 seconds to mimic the normal variation of rivalrous dominance durations.

MEG compatible glasses containing prism lenses were used to fuse the images for all three tasks. While viewing separate right and left eye stimuli with prism glasses was only strictly necessary for the BR task, both the PR and NR tasks were also presented to each eye and fused with lenses for consistency between the three tasks. A rigid black divider was fixed between the nasion and screen to assist with position and stability as well as control of stray light often seen with prism glasses. Fixation marks (center diamond and boarders) were provided to reduce eye movement, and subjects confirmed successful fusion of the images using a preview screen prior to the start of recording.

All recording blocks were preceded by a preparatory alignment screen, and were initiated by the participant via a button press once image fusion was stable. Data collection for each condition consisted of two recording runs. Each run included an alignment screen, 60 seconds of baseline resting state (eyes open with fixation crosshair), followed by 4 blocks of 60-second duration. A short rest was provided between blocks, and a longer rest between runs.

During rivalry tasks, subjects were instructed to respond using two buttons to indicate alternations between percepts. Rather than use a third button for mixed percepts, the subjects were instructed before each recording to use one of the two buttons, in a counter balanced design. In half the trials mixed percepts were reported with the left-eye stimulus dominant button, while in the other half mixed percepts were reported with the right-eye-stimulus dominance button. Events were subsequently marked as either pure (pure dominance percepts) or mixed (mixed plus dominance percepts) based on the instructions for the block. This design was used to equate all three tasks in terms of number of buttons (and fingers used), and still provide an estimate as to the proportion of mixed percepts. An estimate of the proportion of mixed percepts is derived by comparing means between counterbalanced blocks of each response alternative with or without mixed percepts [25]. This strategy also allows close comparison to our previous fMRI studies, e.g., [40].

### Data recording

Brain activity was recorded with a 275-channel CTF/VSM MEG system (CTF MEG, British Columbia, Canada) with a sampling rate of 2500Hz. Subjects’ head shapes, anatomical landmarks and head-position coil locations were digitized using a Polhemus Fastrak system (Polhemus, Vermont, USA). In addition to MEG, bipolar signals were recorded for vertical and horizontal electrooculogram (EOG) and electrocardiogram (ECG). Two photodiodes were placed on the subject screen and used to record exactly the stimulus flicker frequency and phase for each eye separately. These signals were recorded using the analog-to-digital input of the MEG acquisition, with a pull-up resistor applied to the channel. Additionally, empty-room conditions were recorded for 2 minutes on each day to ensure signal quality and for environmental noise modeling in subsequent MEG source imaging.

A T1-weighted anatomical MRI volume was acquired for registration with MEG (1.5T Siemens Sonata). Tissue segmentation and cortical surface extraction were performed with Freesurfer (http://surfer.nmr.mgh.harvard.edu/). For defining individual visual area regions of interest, we used a freely available Dockerized tool (https://hub.docker.com/r/nben/occipital_atlas) to co-register the Wang retinotopic atlas [45] from FreeSurfer's fsaverage brain template to each participant’s brain anatomy.

### Pre-processing

All MEG data analyses were performed with Brainstorm [46] (http://neuroimage.usc.edu/brainstorm). Recordings were cleaned of eye movements and cardiac artifacts using signal space projection (SSP). Bad channels and additional bad segments were marked manually and removed from further processing. A high-pass filter was applied at 0.1Hz to remove the MEG sensors‘ DC offset. Powerline contamination was removed using a sinusoidal removal process at frequencies 60, 120, 180, and 240Hz. We derived distributed source models from an overlapping-spheres head model and the cortically constrained in location and orientation (15,000 elementary current dipoles), weighted-minimum norm estimator (wMNE) available in Brainstorm, all with default parameters [47].

Individual subject mean dominance durations, corresponding to the mean duration of perceptual dominance, were computed using the behavioral data by extracting and averaging the time periods between the button presses, excluding the first period of each block. Mean and standard deviation of subject dominance duration (in seconds) for PR was 3.8 +/-1.6, BR was 2.4 +/- 1.0, and NR was 2.0 +/- 0.6. Subjects were then clustered (median split) in two groups according to their BR mean dominance durations: “Fast Switchers” and “Slow Switchers” (see Results).

Dominant and suppressed frequencies for each trial were defined based on the subjects’ report of a perceptual switch from one percept (suppressed) to another (dominant). For example, in the BR task, when a subject reported a switch from the image presented in the left eye (5Hz) to the one in the right eye (7.5Hz), we labeled that trial with a suppressed frequency of 5Hz and dominant frequency of 7.5Hz. We also labeled instances of pure vs. mixed percepts, based on whether subjects reported transitions from a clear perception of one image (pure) to either another pure perception or a mixed perception of the two images presented (mixed), or vice versa. Statistical inferences were driven based on non-parametric permutations and cluster analysis (1000 randomizations and cluster alpha of 0.05) performed with the corresponding Fieldtrip functions run via Brainstorm [48] (http://www.ru.nl/neuroimaging/fieldtrip).

Finally, the event-related analysis described below was limited to the frequencies that were phase-locked to the stimulus flicker in each eye for all measures. In other words, only evoked activity that oscillated at our fundamental frequencies F1 or F2 (5 or 7.5 Hz) or intermodulation frequency (2.5 Hz) with a constant phase delay with respect to the physical oscillations of the stimuli were extracted from MEG source time series. This strategy isolates the task-specific evoked signals that modulate as a function of perception and avoids the type of sensor contamination that may underlie the appearance of anatomically wide-spread localization of power [32] and coherence modulations [33–34].

### Event related analysis: Fundamental frequencies

**Fig 2:**
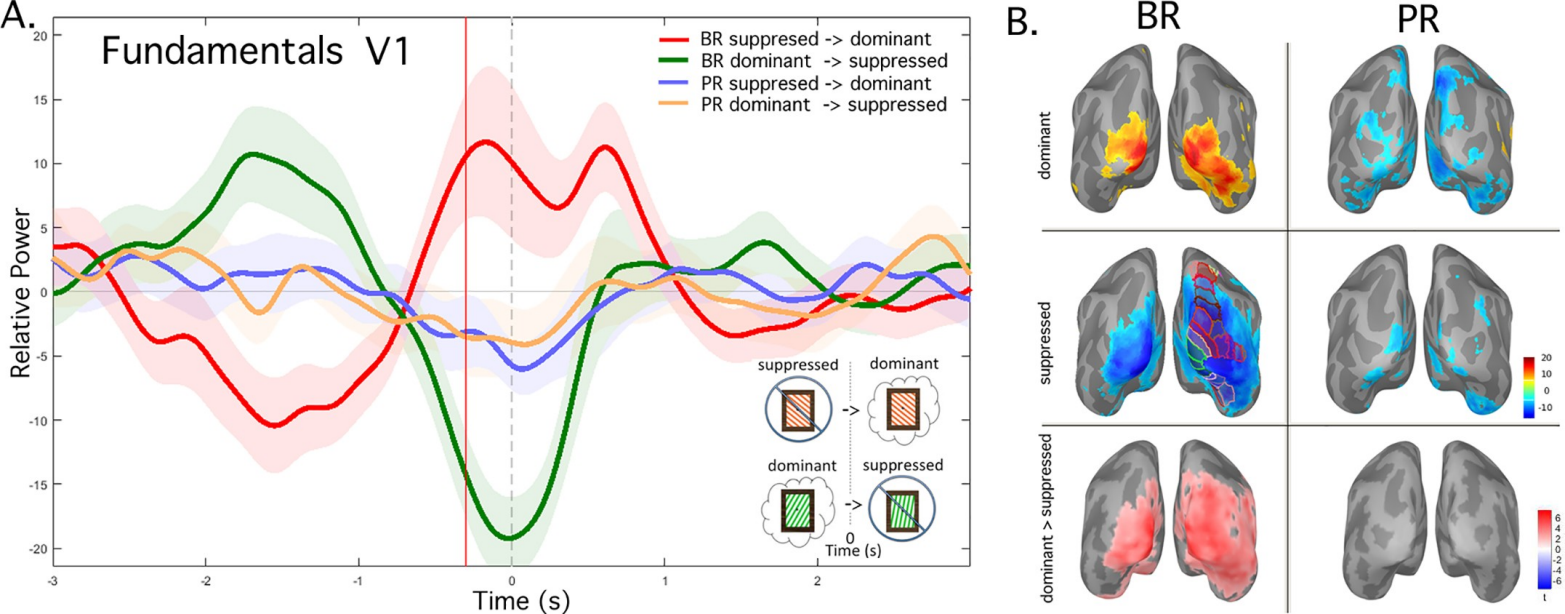
Dominant vs. suppressed fundamental frequency power difference. BR vs. the PR control, group-average (n = 10) A. Power fluctuations over time extracted from right V1. The gray vertical dashed line indicates the time of button press (0 sec) while the red vertical line marks 300 ms prior to the press. For BR, red or green lines show power at the frequency that was perceptually dominant or suppressed after button press, respectively. Pale color fill indicates the standard error of the mean (SEM). For PR control, the same analysis is indicated with blue or yellow lines. B. Dominant (top row) and suppressed (middle row) power was extracted at -300 ms for each condition, for BR (left column) and PR control (right column), and plotted on the inflated cortical surface. Shown also (left column, 2^nd^ row), a probabilistic atlas (Wang, et.al 2015) co-registered to Freesurfer’s fsaverage brain was overlaid on the right hemisphere to provide a reference for visual regions of interest, shown here in multiple colors depicting individual regions. The upper and lower bank of V1 is indicated in bright green in this and subsequent figures. Bottom row shows the regions with a significant difference between the dominant and suppressed power at -300 ms.

For the BR and PR conditions, epochs of (-4,4) seconds around each subject’s reported perceptual alternation were extracted from the 60-second blocks and down-sampled to 120Hz. In addition, only those trials where a single percept was sustained for a minimum of 1 second before and after the alternation were included in subsequent analyses, e.g.,[38]. This criterion resulted in the use of 69% of the trials. Time-frequency decompositions were computed at each cortical source from the modulus of a Morlet wavelet (central frequency = 1Hz with time resolution of 3 seconds) for the fundamental frequencies, 5Hz and 7.5Hz. The dominant frequency power envelope was extracted for each trial (as the squared modulus of the corresponding Morlet coefficient), then averaged across trials. The envelope of the signal power at the suppressed frequency across trials was also derived using the same approach. The average power envelopes were cut to (-3,3) seconds to exclude wavelet edge effects and standardized according the event-related perturbation statistics (Event Related Synchronization/Event Related Desynchronization) across the entire (-3,3) second window. The average dominant and suppressed power measures were extracted at -300ms [34] and compared between the PR and BR conditions (PR: n = 87 +/- 30, BR: n = 138 +/- 54). In addition, the average dominant and suppressed power time series were extracted for the right V1 region (Fig 2).

**Fig 3:**
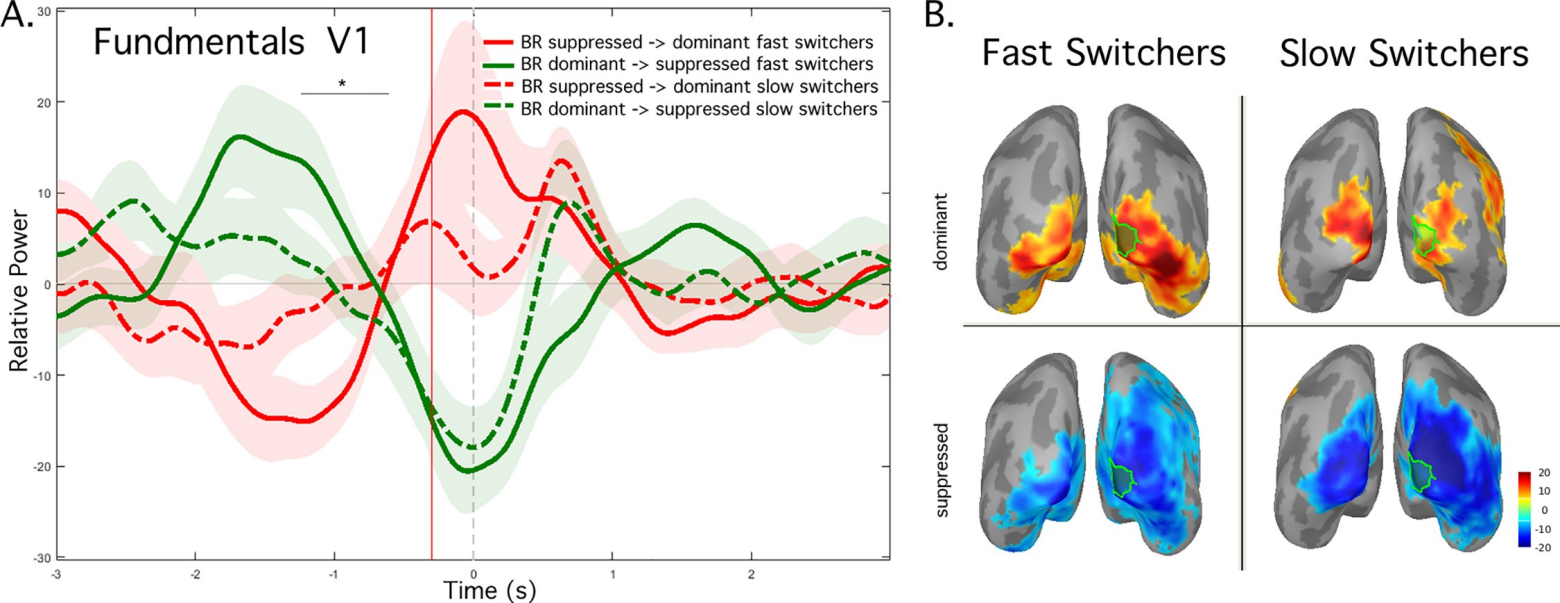
Dominant vs. suppressed fundamental frequency power difference for BR: Fast vs. Slow Switchers, group-average (n = 5 in each group). A. Power fluctuations over time extracted from visual area right V1 for the condition BR for Fast Switchers dominant power (red) and suppressed power (green) and Slow Switchers cortical responses at dominant (dashed red) and suppressed (dashed green) frequencies. Pale color fill indicates the standard error of the mean (SEM). Gray horizontal line with asterisks indicates the brief period when dominant minus suppressed power differed for Fast and Slow Switchers (see text) B. Dominant (top row) and suppressed (bottom row) response maps extracted at -300 ms from button press are visualized on the inflated cortical surface comparing Fast Switchers (left column) and Slow Switchers (right column). Green outlines indicate left and right V1 from probabilistic atlas.

To identify a possible relationship between power fluctuations and Fast vs. Slow Switching behavior, the corresponding dominant and suppressed power measures were group-averaged for Fast Switchers (PR: n = 95 +/- 29 SD; BR: n = 165 +/- 44 SD) and Slow Switchers (PR: n = 79 +/- 32 SD;, BR: n = 111 +/- 52 SD) separately at each cortical source location. The corresponding average time series were extracted for right V1 in the two groups (Fig 3).

### Event related analysis: Intermodulation frequencies

**Fig 4:**
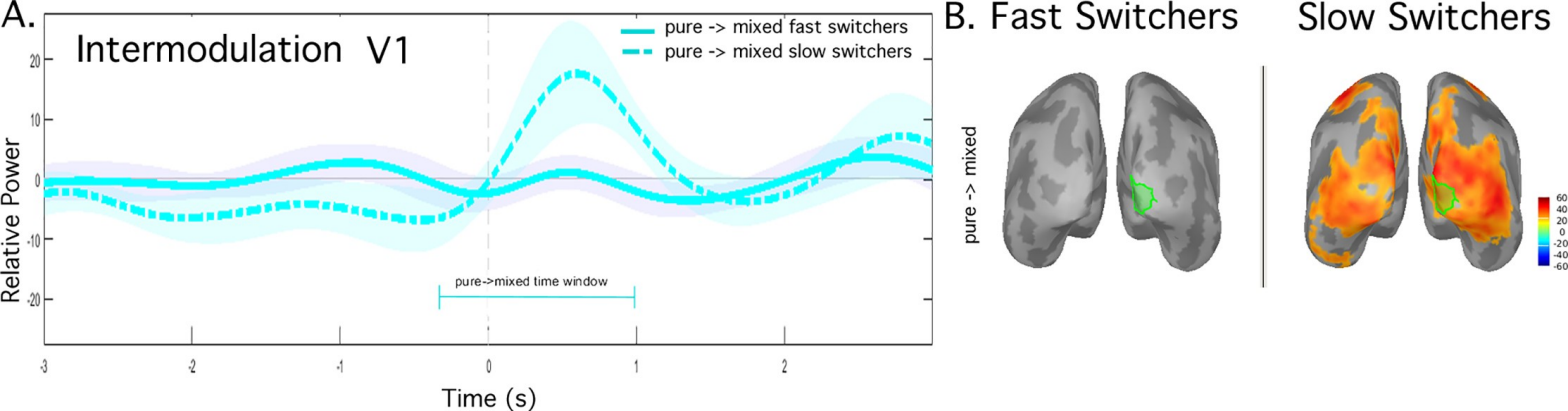
Intermodulation frequency power fluctuations for BR: Fast vs. Slow Switchers, group-average (n = 5 per group). A. 2.5 Hz power over time extracted from visual area right V1 for condition BR and pure to mixed alternations. For Slow Switchers, the dashed cyan time course shows power fluctuations when alternating between epochs with pure dominance (i.e. no-mixed percept) to epochs that sometimes contained a mixed percept. Pale color fill indicates the standard error of the mean (SEM). The equivalent data is shown for Fast Switchers in solid cyan time course. B. Maximum change of 2.5Hz signal power (maximum minus minimum) over the time window, (-300, 1000) ms.

The time-frequency decomposition was derived to identify power changes related to perception at each cortical source at the intermodulation frequency of 2.5Hz (same Morlet mother wavelet as above). Previous reports indicate that mixed percepts are associated with moments of greater responses at the intermodulation frequency [38]. Accordingly, the power envelopes were averaged across trials according to 1]. the two types of events, pure (one eye dominant only) and mixed (one eye dominant or mixed percept), 2]. the two conditions, BR and PR, and 3]. the subject group, Fast Switcher or Slow Switcher. The number of events are as follows: for pure-to-mixed events: BR trials for Fast Switchers (n = 83 +/- 22) and Slow Switchers (n = 54 +/- 27), PR trials for Fast Switchers (n = 47 +/- 15) and Slow Switchers (n = 39 +/- 16); and for trials of mixed-to-pure percepts: BR trials for Fast Switchers (n = 82 +/- 22) and Slow Switchers (n = 57 +/- 25); PR trials for Fast Switchers(48 +/- 15) and Slow Switchers (40 +/- 16). Two different time windows of interest were defined, (-1500, 300) milliseconds for transitions from mixed-to-pure, and (-300,1000) milliseconds for transitions of pure-to-mixed. These time windows were chosen to encompass the epoch either before or after the button press, for mixed-to-pure and pure-to-mixed, respectively [38]. Power fluctuation measures were then computed by taking the difference between the maximum and minimum signal power values observed over the time window of interest at each cortical source location for the different groups (Fig 4).

### Event-related analysis: Power fluctuations related to dominance durations

**Fig 5:**
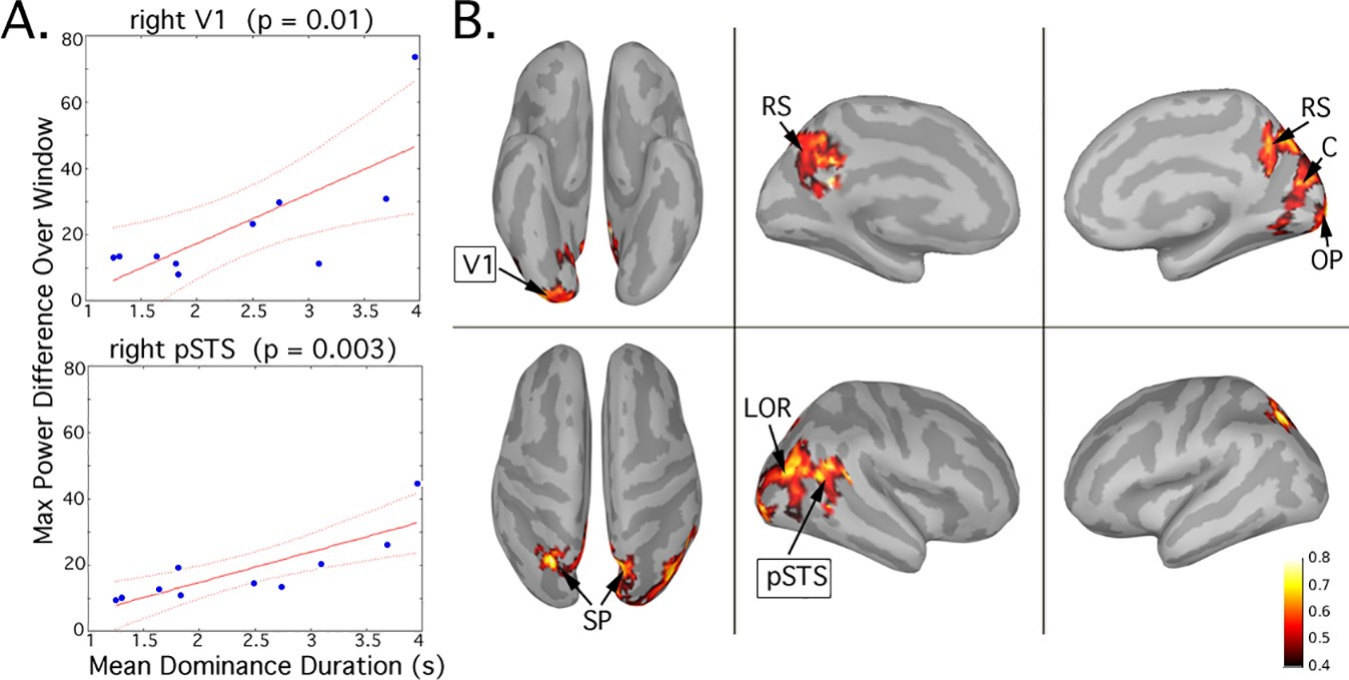
BR mean dominance durations vs. maximum change in 2.5Hz power over the window of interest. A. (top) For right V1, the power envelope at 2.5Hz is extracted from trials classified as pure to mixed, then averaged. The maximum change in power (maximum minus minimum) over the window (-300,1000) milliseconds is computed and correlated with the BR mean dominance durations for the subjects. A. (bottom) The same correlation is shown for right pSTS (posterior superior temporal sulcus) B. Correlations over the entire cortical surface (15,000 sources) with p< 0.05 in clusters > 200 vertices are shown in heat map on the cortical surface.

To examine additional relationships between power fluctuations and individual speed of rivalry, correlations were computed using a linear regression model. The largest change in power at the fundamental frequencies (5Hz and 7.5Hz) in the window preceding a button press was measured; the difference was computed between the maximum value of instantaneous signal power at the dominant frequency and the minimum signal power value at the suppressed frequency over the time window (-300, 1000) milliseconds, for each condition BR and PR. Correlations were computed between the differences and the mean dominance durations of the 10 subjects. To evaluate the signal power at the intermodulation frequency, the 2.5Hz power fluctuation measures were extracted for the two percepts and two conditions; pure-to-mixed (BR (n = 69 +/- 28); PR (n = 43 +/- 15)) and mixed-to-pure trials (BR (n = 70 +/- 26); PR trials (n = 44 +/- 15)). Again, the correlation between the fluctuations and speed of rivalry was evaluated (Fig 5).

## Results

### Speed of perceptual rivalry

As expected, our 10 subjects differed substantially in their individual mean length of dominance duration. For BR the durations were: Fast Switchers (1.2, 1.3, 1.6, 1.8, 1.8 sec) and Slow Switchers (2.5, 2.7, 3.1, 3.7, 4.0 sec). For PR the durations were as follows: Fast Switchers (1.2, 3.0, 3.1, 3.2, 3.2 sec) and Slow Switchers (3.3, 4.3, 4.3, 5.3, 7.2 sec). The mean durations were significantly shorter for BR (2.4 sec) than PR (3.8 sec) (p = 0.007, paired, two-tailed, t = 3.65). There was a near-significant correlation between BR and PR durations, r = 0.62; p = 0.058. The mean percentage of time viewing mixed percepts was 10% for BR and 16% for PR, and the difference was not significant. The mean percentage of time viewing mixed percepts for BR was larger for Slow Switchers (13%) compared to Fast Switchers (6%). There was a weak correlation between BR dominance durations and estimated duration of BR mixed percepts that missed significance (p = 0.084).

### Event-related MEG analysis

Power fluctuations for the dominant and suppressed monocularly tagged frequencies were found to be related to perceptual switches in the BR condition, but not for PR (**Fig 2**). Specifically, the red time course in **Fig 2A** represents the average time course of the suppressed-to-dominant power over the visual area right V1. The green time course represents the average time course of the dominant-to-suppressed power over visual area right V1. Therefore, power of monocular input to V1 was enhanced during perceptual dominance and reduced during suppression. To visualize the extent of this effect across the brain, suppressed-to-dominant and dominant-to-suppressed signal power was extracted at -300 ms prior to button press for each condition and plotted on the inflated cortical surface **(Fig 2B)**. The results for BR were largely confined to the posterior occipital temporal cortex, with no sources in frontal cortex. There was extensive overlap between regions showing dominance-related power enhancement and suppression-related power reduction, although the dominance enhancement was more focused in ventral temporal cortex. The regions showing suppression reduction overlapped with most retinotopic areas in the Wang et al. probabilistic atlas [45] superimposed. In the bottom row, the cortical regions with a significant difference between dominance enhancement and suppression reduction are shown. As expected, for the PR control task, the equivalent time courses of the dominant and suppressed fundamental tagged frequencies before and after each button press was not seen to alternate, shown for PR suppressed-to-dominant (blue) and PR dominant-to-suppressed (yellow)**(Fig 2A)**, nor for the NR control task (not shown). The minimal power fluctuations for PR outside of V1 are shown also in **Fig 2B** for completeness, but it should be recalled that we are tagging the eyes here, not the alternate percepts. No regions showed a significant difference between dominance and suppression. Thus, these results confirm our expectation of minimal monocular competition for PR. In the remaining results we focus primarily on BR.

Next, we repeated the above analysis for BR in the Fast Switchers and Slow Switchers groups. Comparison of the suppressed-to-dominant (red) and dominant-to-suppressed (green) time courses during a perceptual alternation indicated that Fast Switchers (solid lines) showed greater signal power fluctuations at the fundamental frequency than Slow Switchers (dashed lines) (**Fig 3A**). We calculated the difference between dominant and suppressed signals at -300 ms in both subject groups. Although not significant, the difference trended towards a larger response for Fast Switchers. An “omnibus” analysis of all time points in the -3 to 3 s time window indicated only one short window from -1000 to -800 ms in which dominant vs. suppressed signal power was significantly different for Fast vs. Slow Switchers. However, we identified that the effect seems to reflect the difference in the time at which the time courses for dominance-related enhancement and suppression-related reduction intersect. This phenomenon occurred around -800 ms on average for Fast Switchers, vs. -1000 ms for Slow Switchers. Finally, we again found signal power fluctuations at -300 ms to extended beyond the occipital visual cortex (**Fig 3B**). Similar to Fig 2, we found overlap between the cortical sources of dominance-related response enhancement and suppression-related reduction. In addition, the source regions responding at the fundamental frequencies are similar for Fast and Slow Switchers.

We next extended the BR analysis to our intermodulation frequency 2.5 Hz. We plot the time course of cortical response around the button press in right V1 (in cyan) (**Fig 4A)**. Switching events were sorted according to the absence or presence of mixed percepts. Remarkably, for Slow Switchers only, for the pure-to-mixed events, the signal power at 2.5Hz increased after the button press associated with mixed percepts (dashed line). Beyond V1, the cortical regions involved over the -300 to 1000 ms time window further extend over the extrastriate visual cortex (**Fig 4B**).

To formally test the relationship between the length of BR dominance durations and cortical responses in V1, we performed a series of linear regressions between the mean dominance durations and the signal power fluctuations at both the fundamental frequencies (dominant and suppressed) and the intermodulation frequency (pure-to-mixed) The same regressions were performed for PR conditions as an additional control. In all cases the number of observations was 10; the error degrees of freedom was 8. A significant correlation was found only in the case of BR for the intermodulation frequency 2.5Hz during the alternation from pure to mixed percepts (p = 0.04; R^2 =^ 0.44; RMS error = 14.5). The equivalent correlation for PR was not significant (p = 0.57; R^2 =^ 0.04; RMS error = 16.9). When we extracted the time course of fluctuations (difference between signals at dominant and suppressed frequency) for the fundamentals over the same (-300 to 1000 ms) window of interest (see Fig 2) we did not find any such correlation between power and alternation rate for BR (p = 0.62; R^2 =^ 0.03; RMS error = 23.6) or for PR (p = 0.30; R^2 =^ 0.13; RMS error = 13.3).

Given the significant correlation between BR dominance durations and the cortical responses at intermodulation frequency for V1, we further tested this relationship across the entire cortex. The regions where correlations reached significance for the BR condition, pure to mixed alternations, with p< 0.05 in clusters > 200 vertices are shown **Fig 5**. Thus, our source analysis indicates a network of cortical regions. This includes occipital pole, lateral occipital, retrosplenial, and superior parietal cortex in the right hemisphere. Each of these areas also shows a significant correlation when extracted as an ROI and tested separately. There is a trend for significance to actually increase in the cortical areas anterior to V1. The extracted values for right hemisphere are: occipital pole (p = 0.01), precuneus (p = 0.01), retrosplenial (p = 0.01), superior parietal (p = 0.007), lateral occipital regions (p = 0.009), posterior middle temporal gyrus (p = 0.02), and posterior superior temporal sulcus (p = 0.003). In the left hemisphere, no clusters were significant at our standard cluster threshold. However, we did note that when the cluster-size threshold was reduced substantially to 70 vertices, then several regions were visible. When extracted as regions of interest, these left hemisphere clusters also showed significant correlations with dominance durations: occipital pole (p = 0.01), precuneus (p = 0.01), superior parietal (p = 0.006), post-central (p = 0.001), pre-central (p = 0.0006), caudal midfrontal (p = 0.0007). Finally, as a precaution, we examined if the subject with the longest dominance durations could possibly influence the regression analysis as an outlier. We confirmed that all regions in both hemispheres retained a significant correlation between intermodulation power and BR dominance durations after the removal of that subject. This novel result is consistent with recent models of cortical inhibition and binocular rivalry [49, 30], and we examine it further in the Discussion.

## Discussion

In this study we used an MEG steady-state frequency tagging approach in combination with event-related analysis of each subject’s responses during perceptual rivalry. We compared three task conditions that varied in terms of monocular conflict and perceptual bistability. We extracted both fundamental and intermodulation frequencies from the evoked MEG, determined their sources with state-of-the-art methods, and tested if these measures were related to the speed of rivalry. Several original results were obtained. First, we observed that cortical responses at the tagged fundamental frequencies waxed and waned in tandem with cycles of dominance and suppression. Importantly, using stimulus-phase locked signals, we extended this finding far beyond V1 for BR, to include most retinotopic areas in the Wang et al., (2015) atlas [45] used, and especially strong in ventral occipital temporal cortex for dominance-related enhancement. We also confirmed our expectation that no monocular competition occurs for our PR control task. Second, we found that the best predictor of individual dominance durations was the strength of responses at the intermodulation frequency from a novel posterior cortical network. The evidence of stronger 2.5 Hz intermodulation frequency responses in Slow Switchers during mixed percepts are consistent with new models of binocular vision that posit of ocular opponency neurons that increase firing rates as the interocular conflict increases.

### Physiological correlates of rivalry dominance and suppression

We replicated previous observations of the modulation of monocular input frequency responses with rivalry dominance and suppression. However, we precisely extended this observation beyond V1, and results emphasize the ventral occipital temporal cortex. Our data and approach provide a new perspective on the longstanding question: *Where in the brain do stimulus representations alternate during rivalry*? [50,51]. When Logothetis and Leopold reported that the activity of single neurons in monkey inferior temporal cortex correlated more strongly with behavioral rivalry than single neurons in V1, it was surprising to researchers who had modeled rivalry as occurring primarily between monocular neurons in V1. Subsequent work has suggested that the dominant and suppressed images (i.e., the contents of visual awareness) are represented in multiple visual areas, but the issue remains unresolved, and the role of stimulus content is unclear, e.g., [52,3, 53, 54]. fMRI BOLD signals in early visual areas, including V1, have been shown to correlate with the alternating percepts during BR. For example, signal increases when a high-contrast grating is perceived and decreases when a low-contrast grating is perceived, e.g., [55, 2, 3]. Another recent study is especially notable for showing a potential contribution of early visual areas (V3 and V4v) to interocular suppression[20]. In particular, the magnitude of BOLD signal reduction in those areas predicted the individual subject’s strength of perceptual suppression, although the task used was somewhat different from rivalry in order to achieve very strong suppression. This evidence is complemented by other studies that show the higher-tier face or place selective areas in ventral temporal cortex (FFA or PPA) also modulate BOLD signal according to rivalry dominance when face-place dichoptic stimuli are used [3]. One of our present contributions is to show that cortical electrophysiological signals are modulated coherently in all these regions concurrently, even with simple grating stimuli. The precise interplay between feedforward and feedback influences within this hierarchical network is an important topic for future studies.

Another contribution we make here is the ability to separate left and right eye specific responses via two fundamental stimulation frequencies. It should be understood that theoretically fundamental frequencies can be produced by binocular as well as monocular neurons (binocular neurons might produce both fundamentals as well as intermodulation frequencies). Thus, we don’t exclude binocular integration neurons from the regions shown to alternate with perception. Nevertheless, without tagging, and in nearly all previous fMRI studies, it is impossible to know if the effect was driven by monocular or binocular neurons (but see [52]). Exclusively monocular neurons are thought to exist only in the input layers of V1 [56,57]. However, in V1 and beyond, single neurons could be driven more by one eye than the other (e.g. 70% vs. 30%) rather than in an all-or-none fashion. In other words, neurons likely exist along a continuum of binocular integration [58]. The current results suggest that monocularly specific competition may exist throughout several stages of the visual cortical area hierarchy. For example, even in high-tier visual areas such as the FFA and PPA neurons may have both an object-related bias and an eye-of-origin-related bias that supports the experience of binocular rivalry between a face and a house. We also note that in the current study, for any given trial, it is not possible to separate the tagging of eye from the tagging of the stimulus during BR, and there could conceivably be a transfer of tag from eye to stimulus along the hierarchy. On the other hand, we know that the PR control task produced very little fluctuation, showing that the presence of perceptual rivalry per se does alter the fundamentals. We presume that similar modulations according to dominance/suppression might be observed for PR if the stimulus components (i.e., red vs. green gratings) not eyes were differentially tagged, and this is planned in a future study. However, we predict that the magnitude of modulations may be still smaller for PR because of the lack of eye-based competition [40, 59], and consistent with previous findings that binocular rivalry is stronger than other types of rivalry [44, 60].

For the sake of completeness, we also mention a distinct body of literature where binocular rivalry was studied with MEG without employing eye-of-origin tagging. Despite the lack of tagging it may be informative to compare some conclusions [21, 54, 61]. For example, one study utilized an intermittent binocular rivalry paradigm where blank periods were regularly inserted in the stimulus presentation (and stimuli also slowly rotated in both eyes [21]. The result was very slow perceptual alternations that now include extended periods of stabilization of one alternative. The authors used this advantage to compare activity during transitions and stability. Very broadly, they found *transitions* to be more associated with sources in lateral temporal and parietal cortex, and the build-up of perceptual *dominance/suppression* more associated with ventro-lateral occipital-temporal cortex. Our results are certainly in harmony with their view that “perceptual content was thus generally best explained by ventral stream activity.” Moreover, we believe our results may be even more generalizable since our subjects viewed only simple grating stimuli (rather than one eye viewing a face and the other a grating). In other words, the use of face stimuli that could most strongly engage face selective cortex [62] is not required for robust rivalry-related activity in ventral temporal cortex [3].

It is also important to mention that these results provide no evidence for monocular activity in frontal cortex that is both stimulus-driven and alternation related. The generalized involvement of frontal and parietal regions for BR dynamics has been repeatedly suggested and disputed in behavioral and functional neuroimaging studies [63, 61, 64] but see [65,66]. In addition, we suggest that the role of the parietal cortex, while clearly vital to BR, is different from the ventral stream, e.g., [67, 68]. The ventral stream appears to contain the ongoing competition between the rivalrous perceptual alternatives. Alternative roles for the parietal circuits might include attentional control and monitoring or triggering alternations, as discussed later.

Finally, a caveat: Due to the fact that subjects used two buttons (not three) we did not completely separate mixed percepts from periods of relatively pure dominance/suppression. However, our design included two counterbalanced task conditions whereby one button indicated only the pure dominance of one eye, while the other button indicated dominance of the other eye as well as mixed, incomplete dominance periods. We thus calculate the mixed percept proportions by subtracting the mean dominance duration of pure from pure-plus- mixed. We inherited this design from previous detailed psychophysical [69] and fMRI designs [40], and the approximation is still quite informative. In terms of the electrophysiology, we accommodated the limitation by sampling a wide window from -300 to 1000msec around each event. We were therefore able to compare epochs of pure dominance/suppression to epochs that included mixed percepts (Fig 4). Our results may relate to the fact (discussed in [38]) that one study reported increased signaling at intermodulation frequencies near dominance-to dominance transitions [35], but that some mixed percepts were present in those reported transitions that may have produced the increase in intermodulation responses. We believe our comparison is sound, but also acknowledge that replication with a design that precisely isolates mixed percepts would be valuable.

### Physiological correlates of dominance duration

We found that the event-related cortical response at 2.5Hz during binocular rivalry predicted dominance duration in our 10 subjects. Specifically, Slower Switchers, with longer dominance durations, produced greater responses at this intermodulation frequency. Evoked signal power at intermodulation frequencies must be produced by nonlinear combination of each monocular frequency (F1 and F2) in binocular neurons (n*F1 +/- n*F2) [31, 36, 37]. Thus, the theoretical predictions would apply equally to suggest interpretations based on either addition or subtraction of the fundamentals. Indeed, there is evidence for both integration and differencing in the binocular vision literature. Good evidence exists supporting a link between the power of intermodulation frequencies and binocular integration associated with binocular fusion or unattended rivalry [35]. On the other hand, Katyal and colleagues [38,39] considered both possibilities when studying rivalry reports of dominance and mixed percepts, and offered evidence that is consistent with the existence of a differencing operation. In particular, they showed that the magnitude of SSVERs at intermodulation frequencies peaked toward the end of a period of mixed (incomplete) perceptual rivalry dominance, and thus immediately prior to the perceptual resolution of dominance [38]. Consistently, other studies have related the subjective experiences of mixed percepts to periods of high interocular adaptation [30]. Katyal et al. [39] extended these observations, by adapting subjects to a specific class of anticorrelated dichoptic stimuli designed to selectively fatigue mechanisms sensitive to interocular conflict. After adaptation, subjects showed decreased EEG evoked responses at the intermodulation frequency.

When interpreting the current data, we suggest that a very interesting explanation of our correlation of the intermodulation signal with BR dominance durations is that subjects with slow alternation rates have a stronger opponent mechanism needed to drive interocular inhibition and achieve exclusive dominance. This is supported by our estimate that the mean proportion of time perceiving mixed percepts was twice as large for Slow compared to Fast Switchers. Consistently, Katyal et al. [38] reported an effect that was greater for longer periods of mixed percepts. In the same vein, we suggest that the more rapid transitions in the Fast Switchers do not last long enough to produce detectable responses in conflict detecting neurons. While there is ample precedence for opponent processing in other domains of vision, e.g. color vision, the potential role in binocular vision, including stereopsis, has not received much attention until quite recently [30, 70, 71], despite the known disparity sensitivity of single neurons, e.g., [72]. Instead, correlation computations, and winner-take-all approaches have been major theories. The presence of such interocular differencing channels has been elegantly demonstrated psychophysically, and explained computationally as an efficient coding technique [73–75].

More specifically, as proposed by Said and Heeger [30], these opponent neurons would increase in firing rate as the dichoptic difference increases. This would be achieved via excitatory input from (e.g., dominant) ‘eye1’ neurons, and inhibitory input from (e.g., suppressed) ‘eye2’ neurons, (thus taking a difference). The output would be inhibitory onto ‘eye2’ neurons and thus add more inhibition when they are suppressed. In this way, opponent neurons supplement the inhibition of the suppressed alternative beyond what would be achieved by the standard model of competitive mutual inhibition between the monocular pools of neurons. This might be similar to the presumably cooperative effects of competition between binocular neurons (based on partial monocular bias, or on stimulus features), e.g., [29]. Finally, we note in passing that a distinctive signature of the efficient encoding theory is a higher gain applied to the difference channel than the summation channel, and this has not yet been demonstrated physiologically [71]. Despite the appeal of the opponent differencing theory, we certainly do not rule out a contribution to evoked intermodulation frequencies from binocular integration neurons, especially for higher-tier visual areas and association cortex where substantial numbers of monocular neurons are not expected. Lastly, the literature we refer to so far uses the concept of binocular combination to refer to retinotopically local dichoptic difference. However, these computations might occur more globally in higher-tier visual areas, e.g., [35].

In a previous fMRI study of binocular rivalry [40], we found that the strength of BOLD signal measured in V2 and V3 was correlated with mean dominance duration across subjects (greater in slow switchers). We offered an interpretation based on the balance of excitation and inhibition in visual cortex, but this is difficult to assess with fMRI. We also recently found with MEG that the peak frequency of the evoked gamma band response (GBR) to simple nonrivalrous grating stimuli was greater in slow switchers. GBR frequency may also be correlated to the local concentration of inhibitory neurotransmitter GABA in V1, as measured in humans by MRI spectroscopy [22]; but see [23]. Overall, the literature does leave us with the possibility that evoked GBR frequency is a non-invasive proxy measure of at least *one type* of resting inhibition in V1, and that GABA-mediated inhibition tends to be higher in slow switchers [25, 26]. We predicted in the previous paragraph that slow switchers have a stronger opponent mechanism leading to a stronger response at 2.5 Hz. Although that putative mechanism requires both excitatory and inhibitory synapses, these opponent neurons primarily enhance the inhibition experienced by suppressed neurons [30]. Taken together, these findings *suggest* that abundant GABA in slower switchers might contribute to both higher evoked GBR frequency and stronger opponency-based inhibition. However, we do relate the gamma literature to the current findings with caution. Please note that rivalry oscillations occur on a vastly slower time scale than that of the typical cycle of gamma oscillations, and the relationship between gamma and inhibition has only been suggested for V1, and this remains a contentious finding [22–24]. Our correlation between dominance durations and intermodulation SSVEP was found in several other cortical regions discussed below.

Several regions in which the power of the intermodulation frequency signal correlates with dominance durations were observed, allowing us to predict individual differences. This network consisted of occipital pole, lateral occipital cortex, posterior STS, retrosplenial, and superior parietal cortices. We find it notable that our own previous fMRI study comparing the areas activated by either a depth discrimination task or a rivalry task using the same stimuli highlighted similar areas including right lateral occipital cortex, right posterior STS, and bilateral retrospenial cortex [76]. These regions are briefly discussed in turn: 1. The lateral occipital cortex has been strongly associated with figure/ground organization, visual segmentation and grouping, e.g. [59, 77]. 2. Single neuron responses in the macaque STS have been found to be strongly modulated by rivalry, although it is not clear that this area is a direct homologue to human pSTS [51]. 3. The retrosplenial cortex is considered part of a network of brain regions subserving seemingly diverse cognitive functions, including episodic memory, spatial navigation [78] as well as the so-called *default mode network*, e.g. [79–81]. It has also been shown to be involved in tasks requiring endogenous monitoring of conflict, as well as during rivalry [82]. In the neighboring precuneus region neurons are affected by the predictability of visual information over long time scales (12 s), suggesting a role in the accumulation of input over time [83]. We speculate here that binocular rivalry might invoke cortical circuits that deal with conflict and conflict accumulation in an increasingly abstract way. Perhaps rivalry tasks share cortical mechanisms with cognitive tasks such as the Stroop task, e.g., the retrospenial/posterior cingulate, due to accumulation of conflict information that plays a modulatory role.

Finally, we also found the correlation between dominance durations and intermodulation frequency to be significant in parts of the right superior parietal cortex. These results are quite consistent with TMS studies that implicated a causal role for right parietal areas in the modulation of perceptual states during rivalry alternations. This includes two separate areas in which TMS has been found to affect rivalry rates in different directions—either increasing or decreasing rates. Moreover, for binocular rivalry, our observed activity is stronger in the right hemisphere than in the left—which is also compatible with the lateral specificity observed by [63, 68, 84–87]. We further note that TMS over parietal cortex has also been reported to disrupt alternation to one but not the other rivalrous alternative [88]. This was explained by a model of binocular rivalry that invokes the ideas of interhemispheric switching, and coupled oscillators, or a more subtle theory of greater contribution of certain regions (e.g., parietal) of one hemisphere (e.g. right) to one perceptual alternative than the other [89]. These ideas also helped explain why very slow rivalry might be associated with psychiatric disorders like bipolar disorder in association with slow prefrontal interhemispheric switch rhythms, and alpha EEG prefrontal asymmetries [14]. Subsequent studies indicate that (healthy) slower switchers tend to show slower alpha oscillations during rivalry, but faster gamma oscillations in V1 [25, 90]. Future MEG studies may provide some support for a common factor that influences multiple oscillations, such as phase-amplitude-coupling between different frequencies, e.g. [91]. It is possible that such a factor could ultimately help explain individual differences.

In summary, we used event-related SSVERs with dichoptic flicker to isolate eye specific and binocular processes during perceptual rivalry. The strength of signal at each extracted frequency was then related to the moments of dominance alternation. In contrast to the results emphasized by many recent fMRI and TMS studies of binocular rivalry, our MEG imaging approach provides a striking display of the role of ventral temporal cortex. We propose that the entire ventral occipital-temporal ‘stream’ is relatively isolated in the high degree to which it manifests the synchronous neural networks that competitively alternate to represent the currently selected percept. The ventral stream (involved in identification and categorization of visual objects) is thus a primary substrate for the competition between the populations of neurons that represent the ongoing perceptual resolution of ambiguity. Secondly, we have provided novel evidence in support of opponency mechanisms in binocular rivalry by predicting dominance durations with the nonlinear binocular mechanisms that underlie SSVER at intermodulation frequencies. We suggest that exploring these individual differences is an important way to further refine the power of our explanatory models of binocular vision. These fundamental micro-circuits of visual cortex are isolated during rivalry, but are also used constantly in natural vision. Moreover, this approach provides a powerful window onto cortical processing at multiple scales. The multi-region network we isolated overlaps with the so-called Default Mode Network that is associated broadly with both normal and abnormal interoceptive integration and monitoring. Thus, this work points to a possible future bridge between the domains of sensory and cognitive neuroscience that might ultimately extend to psychiatry.
